# Overexpression of colorectal cancer oncogene CHRDL2 predicts a poor prognosis

**DOI:** 10.18632/oncotarget.14039

**Published:** 2016-12-20

**Authors:** Jian Sun, Xuan Liu, Hong Gao, Long Zhang, Qing Ji, Ziyuan Wang, Lihong Zhou, Yan Wang, Hua Sui, Zhongze Fan, Qi Li

**Affiliations:** ^1^ Interventional Cancer Institute of Integrative Medicine & Putuo Hospital, Shanghai University of Traditional Chinese Medicine, Shanghai 200062, China; ^2^ Department of Medical Oncology, Shuguang Hospital, Shanghai University of Traditional Chinese Medicine, Shanghai 201203, China; ^3^ Cancer Institute of Traditional Chinese Medicine & Longhua Hospital, Shanghai University of Traditional Chinese Medicine, Shanghai 200032, China

**Keywords:** colorectal cancer, BMP, CHRDL2, proliferation, apoptosis

## Abstract

Bone morphogenetic proteins (BMPs) both promote and suppress tumorigenesis, and multiple BMP antagonists reportedly contribute to cancer progression. In this study, we demonstrated that the BMP antagonist Chordin-like 2 (CHRDL2) is upregulated in colorectal cancer (CRC) tissues, and that CHRDL2 levels correlate with clinical features of CRC patients, including tumor size, TNM staging, and tumor differentiation. In addition, survival rate and Cox proportional hazards model analyses showed that high CHRDL2 levels correlate with a poor prognosis in CRC. Moreover, CHRDL2 promoted CRC cell proliferation *in vitro* and *in vivo*, perhaps through up-regulation of Cyclin D1 and down-regulation of P21. Co-immunoprecipitation assays showed that CHRDL2 bound to BMPs, which inhibited p-Smad1/5, thereby promoting CRC cell proliferation and inhibiting apoptosis. These results suggest CHRDL2 could serve as a biomarker of poor prognosis in CRC, and provide evidence that CHRDL2 acts as an oncogene in human CRC, making it a novel potential therapeutic target.

## INTRODUCTION

Colorectal cancer (CRC) is the third most common malignant tumor. Approximately 608, 000 people die of colorectal cancer each year, accounting for 8% of the total number of deaths worldwide [1]. The 5-years overall survival (OS) rates after initial colorectal surgery in CRC patients is negatively correlated with the degree of differentiation, depth of invasion, TNM stages, and lymph node metastasis [2, 3]. Recent studies showed that changes in cytokine signaling networks, such as transforming growth factor (TGF-β), tumor necrosis factor (TNF), and interleukin (IL), contribute to tumor progression CRC [4].

The TGF-β superfamily of proteins regulates tumor cell differentiation, proliferation, migration, and apoptosis [5–7]. The TGF-β superfamily is a set of multifunctional proteins including TGF-β, Bone Morphogenetic Proteins (BMPs), Growth Differentiation Factors (GDFs), Glial-derived Neurotrophic Factors (GDNFs), Inhibits, Nodal, Activins, Lefty, and Mülllerian Inhibiting Substance (MIS). This superfamily also includestwo kinds of receptors, which are intracellular signaling mediators for the TGF-β superfamily [5]. Type I receptors include Activin and Nodal, whose signalsaretransduced through Smad2 and Smad3. Type II receptors include BMPs and GDFs, which transduce signals through Smad1, Smad5, and Smad8 [8]. Extracellular antagonists tothe TGF-β superfamily are secretory proteins that bind to TGF-β superfamily ligands to interfere with their function. These antagonists, including Noggin, Follistatin, Sclerostin, Chordin, Cerberus, and Gremlin, downregulate signaling by the TGF-β superfamily and promote tumor development. By antagonizing BMP2, Noggin accelerates the proliferation of gastric cancer cells [9]. Follistatin, an antagonist of Activin, promotescancer cell survival [10]. Gremlin-1, an antagonist of BMPs, induces cell migration, invasion, and proliferation [11].

Bone morphogenic proteins (BMPs) belong to the TGF-β superfamily and help to control colonic epithelial cell renewal, proliferation, and differentiation, and also possess potent antitumor activity [12]. Signaling by BMPs is triggered by BMP ligands binding to BMP receptors (BMPRs) followed by Smad1/5/8 activation. Activation of BMP signaling can promote apoptosis of mature colonic epithelial cells, thereby maintaining the dynamic balance of colonic epithelial cell proliferation and differentiation [13, 14]. Extracellular antagonists such as Noggin can inhibit BMP signaling in colon stem cells, which is necessary for the self renewal and proliferation of intestinal crypt stem cells [13, 15]. Recent research has shown that BMP signaling is lost or blocked in CRC, stemming from mutations in the *BMPR* and *Smad* genes and resulting in low expression of BMPs as well as high-expression of BMP antagonists [16–19].

Chordin-like 2 (CHRDL2) is an antagonist of BMPs that prevents them from interacting with their cognate cell surface receptors [20]. Although a previous study showed higher levels of *CHRDL2* mRNA in breast cancer [21], CHRDL2's role in tumorigenesis remains largely unknown. Here, we measured CHRDL2 levels in human CRC tissue to investigate potential correlations between CHRDL2 expression and CRC clinicopathologic features as well as patient prognosis. We also investigated CHRDL2's role in CRC cell cycle progression. We showed that CHRDL2 was overexpressed in CRC, and this correlated with a low survival rate and poor prognosis. Furthermore, we showed that overexpression of CHRDL2 in CRC cell lines accelerated cell growth *in vitro* and promoted tumorigenesis *in vivo*, whereas knockdown of CHRDL2 showed the opposite effect. We also showed that CHRDL2 could block the activation of Smad1/5, as well as proliferation-inhibition and apoptosis induction by BMP2 in CRC cells. Our data suggest that CHRDL2 acts as an oncogene in CRC and might be a potential therapeutic target for CRC.

## RESULTS

### CHRDL2 variant I is the major gene type in CRC

Ten *CHRDL2* variants were previously identified in various tissues [22]. To study the gene structure of CHRDL2 in CRC, the CHRDL2 gene open reading frame sequences of five pairs of colorectal cancer tissue and their matched normal tissues (N, normal tissue, T, tumor tissue) were amplified by RT-PCR. The PCR products were separated and visualized by electrophoresis (Supplementary Figure S1A): Four lanes (T1, T3, T4, N1) were found to have 4 product bands (B1, B2, B3, B4), two samples (T2, N3) having a major band (B1), two samples (T5, N5) a weak band (B2), and two lanes (N2, N4) no bands (B2). PCR products were subsequently isolated, purified, subcloned and sequenced.

The CHRDL2 RT-PCR product (B1) was identified to be the CHRDL2 variant I (GenBank Acc. No. AY279090.1), and B2 and B4 were non-specific sequences while B3 was identified as a new CHRDL2 (BNF1) variant (AEV56635.1). As shown in Supplementary Figure S1B, the amino acid sequence of B1 is the CHRDL2 variant I (AY279090.1) while 293 amino acids were deleted in B3 (AEV56635.1). CR2 and CR3 are located in such deleted area. These data revealed that CHRDL2 variant I was the major CHRDL2 gene type in CRC tissues while the new CHRDL2 (BNF1) variant (AEV56635.1) was expressed at low levels in colorectal cancer and normal tissue. CR2 and CR3 deletion may cause the inactivation of CHRDL2 gene; therefore, we selected the CHRDL2 variant I gene for further functional studies.

### Higher CHRDL2 levels in CRCs are correlated with clinical features and pathologic parameters of CRCs patients

**Figure 1:**
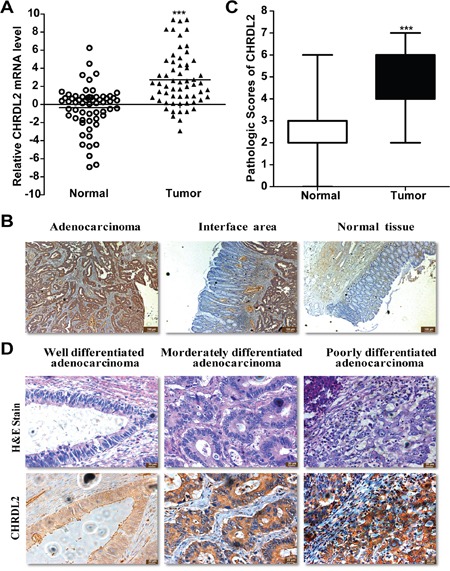
CHRDL2 expression is significantly increased in CRC tissues **A**. Quantitative RT-PCR of *CHRDL2* mRNA expression levels in 60 paired human CRCs and normal tissues. *CHRDL2* expression levels were normalized by those of GAPDH. Data were calculated from triplicate measurements. T, tumor samples. N, matched normal tissues. **B**. Representative images of immunohistochemical staining of CHRDL2 in 125 human CRC patients. **C**. Pathologic scores of CHRDL2 in CRC tissues. The scale bar represents 100 μm. Values were expressed as mean ± SD. (*** *p*<0.001). **D**. Immunohistochemistry images for CHRDL2 in three differentiated degree human CRC samples. Hematoxylin-eosin (H&E) stain of the same samples is also shown. The scale bar represents 20 μm.

To investigate the expression status of *CHRDL2* gene in CRCs, we quantified mRNA levels of *CHRDL2* by Quantitative RT-PCR (QRT-PCR) in 60 pairs of primary tumors and their matched adjacent normal tissues. The result showed that *CHRDL2* mRNA levels were markedly higher in CRC samples than in their adjacent normal tissue counterparts (*P*<0.001; Figure 1A). Immunohistochemistry analyses further confirmed the upregulation of CHRDL2 in CRC samples. Even in interface areas, CHRDL2 was still overexpressed in tumor areas compared with normal areas (Figure 1B). The expression of CHRDL2 protein was higher in colorectal adenocarcinoma and in the CRC samples than in their adjacent normal tissue counterparts (*P*<0.001) (Figure 1C). Figure 1D showed that CHRDL2 levels are higher in poorly- and moderately-differentiated adenocarcinoma than in well-differentiated adenocarcinoma. To compute the correlation between CHRDL2 expression and clinical and pathological parameters, the samples were grouped into high CHRDL2 level (staining immunohistochemical score>4) and low CHRDL2 level (staining immunohistochemical score≤4) groups. The correlations between CHRDL2 expression and clinical and pathological characteristics are presented in Table 1. High CHRDL2 levels correlated with larger tumor size (*P*<0.01), later TNM staging (*P*<0.01), and poor tumor differentiation (*P*<0.01). However, there was no correlation between CHRDL2 protein expression and tumor location, gender, depth of infiltration, lymph node metastasis, or age.

**Table 1:** Correlation of CHRDL2 expression with clinicopathologic features in CRC tissues

Clinicopathologic features	Expression of CHRDL2 protein(IHC)			
	Total cases	High expression	Low expression	*P* values
Age(years)				0.911
<60	76	38	38	
≥60	49	24	25	
Gender				0.792
Female	59	30	29	
Male	66	32	34	
Tumor diameter(cm)				0.009**
≤5	71	28	43	
>5	54	34	20	
Tumor location				0.858
Colon	69	35	34	
Rectum	56	27	29	
Depth of infiltration				0.385
Submucosa, musculeris propria	28	15	13	
Subserosa	66	29	37	
To the surrounding tissue	31	18	13	
Histologic type				0.023*
Well differentiated	56	22	34	
Morderately differentiated	49	25	24	
Poorly differentiated	20	15	5	
TNM staging				0.002**
I/II	81	32	49	
III/IV	44	30	14	
Lymph node metastasis				0.916
YES	45	26	19	
NO	80	47	33	

NOTE: *p* values were derived by using Chi-square test. (* *p*< 0.05. ** *p*< 0.01).

### Higher CHRDL2 levels correlate with poor prognosis in CRC

**Figure 2:**
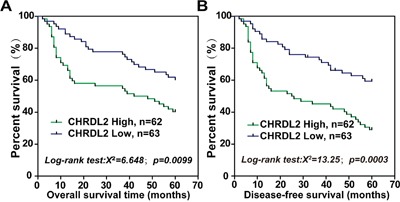
CHRDL2 is a potential predictor of poor prognosis in CRC patients Kaplan–Meier curves displaying **A**. disease-free survival and **B**. overall survival in patients with primary CRC. According to pathologic scores, CRC patients were divided into high CHRDL2 expression (>4) and low CHRDL2 expression (≤ 4) groups.

We used the Kaplan–Meier analysis to analyze CRC patient survival and detect any potential correlations between survival and CHRDL2 levels. In this study, during the follow-up time of 60 months 46 patients with CRC died from CRC-related disorderswhile 8 patients died from other causes and were thus excluded. In addition, another 12 patients with data missing were also excluded during the follow-up time. The Kaplan–Meier analysis results for OS and DFS are displayed in Figure 2A and 2B respectively. As shown in Figure 2A and 2B, the log-rank test indicated that CRC patients with high CHRDL2 scores had a shorter overall or disease-free survival than CRC patients with low CHRDL2 scores (log-rank value 8.45 and 7.398; *P*<0.01 and *P*<0. 01, respectively).

Next, we used univariate and multivariate Cox proportional hazards model analysis to analyze the effect of CHRDL2 on OS and DFS. Clinical and pathological characteristics including age, gender, tumor location, tumor diameter, TNM stage, depth of infiltration, histologic type and lymph node metastasis were considered to adjust the hazard ratio (HR). The univariate analysis revealed that tumor diameter, TNM stage, histologic type, and tumor CHRDL2 expression were prognostic indicators for OS, while multivariate analysis excluded depth of infiltration (Table 2). Regarding DFS, the univariate and multivariate analyses revealed that tumor diameter, TNM stage, histologic type, and tumor CHRDL2 expression were independent prognostic factors (Table 3).

**Table 2:** Univariate and multivariate analysis of overall survival in CRC patients

Variables	No. of cases.	Univariate analysis			Multivariate analysis		
		HR ratio	95%CI	P-value	HR ratio	95%CI	P-value
Age(years)		1.004	0.985-1.204	0.664			
<60	76						
≥60	49						
Gender		1.007	0.625-1.853	0.79			
Female	59						
Male	66						
Tumor diameter(cm)		2.157	1.252-3.715	0.006**	2.158	1.245-3.741	0.006**
≤5	71						
>5	54						
Tumor location		0.664	0.377-1.169	0.156			
Colon	69						
Rectum	56						
Depth of infiltration		1.185	0.815-1.723	0.373			
Submucosa, musculeris propria	28						
Subserosa	66						
To the surrounding tissue	31						
Histologic type		1.518	1.053-2.189	0.025*	1.696	1.147-2.506	0.009**
Well differentiated	56						
Morderately differentiated	49						
Poorly differentiated	20						
TNM staging		2.234	1.297-3.848	0.004**	2.489	1.481-4.399	0.002**
I/II	81						
III/IV	44						
Lymph node metastasis		1.609	0.973-2.659	0.064			
NO	85						
YES	40						
CHRDL2 expression		2.231	1.275-3.905	0.005**	2.139	1.207-3.791	0.009**
Low	63						
High	62						

NOTE: *p* values were derived by using Cox proportional hazards regression model. (* *p*< 0.05. ** *p*< 0.01).

**Table 3:** Univariate and multivariate survival analysis of disease-free survival in CRC patients

Variables	No. of cases.	Univariate analysis			Multivariate analysis		
		HR ratio	95%CI	P-value	HR ratio	95%CI	P-value
Age(years)		1.009	0.991-1.027	0.349			
<60	76						
≥60	49						
Gender		0.911	0.554-1.498	0.713			
Female	59						
Male	66						
Tumor diameter(cm)		0.566	0.3629-0.8826	0.012*	1.719	1.049-2.816	0.019*
≤5	71						
>5	54						
Tumor location		0.996	0.609-1.628	0.987			
Colon	69						
Rectum	56						
Depth of infiltration		1.055	0.749-1.485	0.761			
Submucosa, musculeris propria	28						
Subserosa	66						
To the surrounding tissue	31						
Histologic type		1.61	1.155-2.245	0.005**	1.615	1.138-2.293	0.007**
Well differentiated	56						
Morderately differentiated	49						
Poorly differentiated	20						
TNM staging		1.716	1.055-2.791	0.029*	1.849	1.119-3.057	0.007**
I/II	81						
III/IV	44						
Lymph node metastasis		1.811	1.099-2.983	0.02*			
NO	85						
YES	40						
CHRDL2 expression		1.956	1.188-3.221	0.008**	1.956	1.188-3.221	0.008*
Low	63						
High	62						

NOTE: *p* values were derived by using Cox proportional hazards regression model. (* *p*< 0.05. ** *p*< 0.01).

### CHRDL2 expression in CRC cells

**Figure 3:**
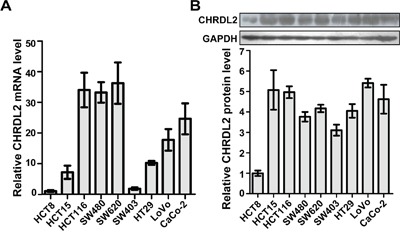
The mRNA and protein expression of CHRDL2 in nine CRC cell lines **A**. *CHRDL2* mRNA expression was measured by QRT-PCR in 9 human CRC cell lines (HCT8, HCT15, HCT116, SW480, SW620, SW403, HT29, LoVo, and CaCo-2). The mRNA level was normalized to 1 for HCT8. **B**. Protein levels of CHRDL2 in human CRC cell lines were measured by western blot. GAPDH was used as a loading control. The CHRDL2 protein level was normalized to 1 for HCT8.

We filtered colorectal cancer cell lines by examining CHRDL2 levels using QRT-PCR and western blotting. As shown in Figure 3A and 3B, the ileocecal colorectal adenocarcinoma HCT8 and colon adenocarcinoma SW403 cell lines showed a relatively low expression of CHRDL2 while SW480 (colon adenocarcinoma), SW620 (colon adenocarcinoma) and HT29 (colon adenocarcinoma) had moderate CHRDL2 levels. On the other hand, HCT15 (colorectal cancer), HCT116 (colon adenocarcinoma), LoVo (colon adenocarcinoma) and CaCo-2 (rectal adenosquamous) exhibited higher CHRDL2 levels. Finally, two CRC lines, CHRDL2 high-expressing HCT116 and CHRDL2 low-expressing HCT8 cells were selected from nine CRC cell lines for further studies.

### CHRDL2 promotes proliferation in CRC cells *in vitro*

**Figure 4:**
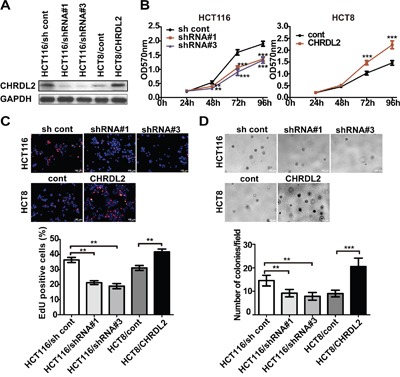
The effect of CHRDL2 on cell proliferation of CRC cells **A**. Corresponding protein expression of CHRDL2 in lentivirus-transfected CRC cells were measured by western blot. HCT8 was stably transfected with either empty vector or CHRDL2 expression vector. HCT116 was stably transfected with either control vector or CHRDL2-shRNA vector. **B**. The MTT assay showed that the inhibition of CHRDL2 suppressed the proliferation of the HCT116 cell lines, while the overexpression of CHRDL2 increased the proliferation of the HCT8 cell lines *in vitro*. (**p<0.01, ***p<0.001). **C**. EdU staining showingthat HCT116 cell proliferation decreases when CHRDL2 is silenced, while HCT8 cell proliferation increases when CHRDL2 is overexpressed. Six views were selected randomly in each slide, the percentage of EdU-positive cells in total cells of the field were quantified. **D**. The proliferation ability of the cells was observed by soft agar colony formation. Mean values from three independent experiments were taken as the results. (**p<0.01, ***p< 0.001). The histograms show mean ± SE of colony numbers. (**p<0.01, ***p<0.001).

Our analyses of clinical samples suggested potential involvement of CHRDL2 in the development of CRC. However, the underlying mechanism remains unclear. Transfected by lentivirus vector, *CHRDL2* gene over-expressing HCT8 cells and *CHRDL2* gene knock-down HCT116 cells were established. We used western blotting to confirm the overexpression or silencing of CHRDL2 in stable cell lines (HCT8/cont, HCT8/CHRDL2, HCT116/sh cont, HCT116/shRNA#1 and HCT116/shRNA#3) (Figure 4A). We discarded the HCT116/shRNA#2 clone due to its lower silencing efficiency. Given that CHRDL2 levels were positively correlated with tumor size, we examined the cell growth potential of the aforementioned clones. As shown in Figure 4B, cell growth was enhanced by the overexpression of CHRDL2 in HCT8 cells while attenuated by the silencing of CHRDL2 in HCT116 cells. EdU can be incorporated into DNA during active DNA synthesis, which can measure cell growth. EdU staining fluorescence images showed that the EdU positive cells (nuclei were stained red) ratio of HCT116/shRNA#1(#3) was lower than that of HCT116/sh cont, and the EdU positive cells ratio of HCT8/CHRDL2 was higher than that of HCT8/cont (Figure 4C). We next evaluated the effect of CHRDL2 on anchorage independent cancer cell growth (soft agar colony formation). As shown in Figure 4D, the number of cancer cell colonies was significantly increased in CHRDL2 overexpressing HCT8/CHRDL2 cells compared with control HCT8/cont cells. On the other hand, the number of colonies decreased in CHRDL2-silenced HCT116/shRNA cells compared with control HCT116/cont cells.

### CHRDL2 increased tumorigenicity of CRC cells *in vivo*

**Figure 5:**
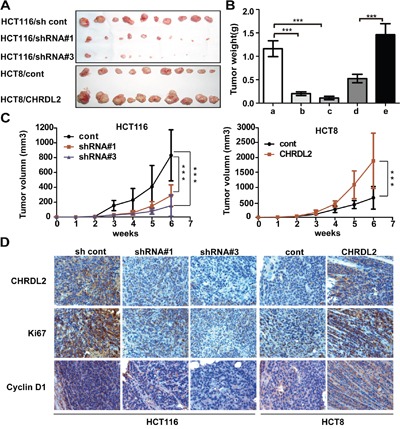
The effect of CHRDL2 on tumor growth of subcutaneous xenograft of CRC cells in nude mice **A**. Lentivirus-transfected CRC cells (HCT116/sh control, HCT116/shRNA#1, HCT116/shRNA#3, HCT8/cont, HCT8/CHRDL2) were used for *in vivo* xenograft tumor growth assay, with 2×10^6^ cells being subcutaneously injected into a flank of nude mice. **B**. The histograms show the weight of xenograft tumors (**P<0.01, ***P<0.001). **C**. Cure graph showing the volume of xenograft tumors forevery week during6 weeks (**P<0.01, ***P<0.001). **D**. IHC staining of CHRDL2, Ki67, and cyclin D1 expression in subcutaneous xenograft tumor. The scale bar represents 100 μm.

Having demonstrated *in vitro* that CHRDL2 enhanced CRC cell growth, we next CHRDL2 affected *in vivo* xenograft tumor growth of CRC HCT116 and HCT8 cells. To this end, we flank-injected nude mice with the aforementioned CRC cells. Consistent with our *in vitro* proliferation results, the volume and weight of tumors from HCT116/shRNA xenografts were lower compared with controls, while HCT8/CHRDL2 tumors had larger volume and weight than the controls (Figure 5A-5C). These data suggested that CHRDL2 promoted tumorigenicity of the CRC cells. We then embedded xenograft tumor tissues in paraffin for immunohistochemistry analyses. CHRDL2, ki67 and cyclin D1 antibodies were used for immunohistochemistry analyses. Ki67 staining was used as a cell proliferation marker, and cyclin D1 was used as a cell cycle marker. As shown in Figure 5D, upregulation of CHRDL2 increased immunostaining of ki67 and cyclin D1 in grafted HCT8 cells while downregulation of CHRDL2 decreased immunostaining of ki67 and cyclin D1 in grafted HCT116 cells. Taken together, in agreement with our experiments *in vitro*, our data from *in vivo* experiments also indicated that CHRDL2 increased tumorigenicity of CRC cells.

### CHRDL2 blocked Smad1/5 signaling via binding to BMPs

**Figure 6:**
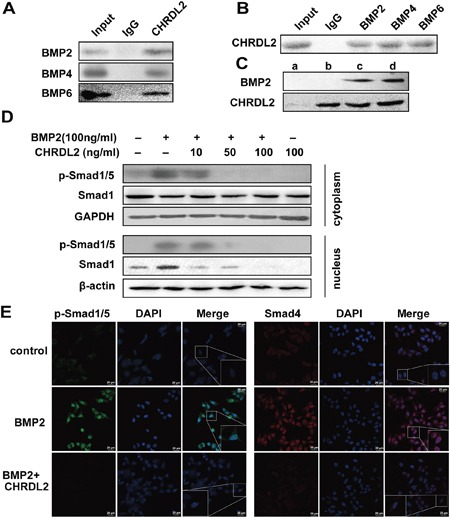
CHRDL2 binds to BMP2 and inhibits the phosphorylation of Smad1/5 **A**. Coimmunoprecipitation (co-IP) of CHRDL2 and BMPs. HCT116 cell culture media were performed using anti-CHRDL2 and control (mouse IgG) antibodies. Immunoprecipitates and culture media were subjected to western blot analysis using anti-BMP2, anti-BMP4, and anti-BMP6 antibodies. **B**. Culture media were performed using anti-BMP2, anti-BMP4, anti-BMP6 and control (rabbit IgG) antibodies, and western blot analysis using anti-CHRDL2 antibody. **C**. Co-IP of recombinant CHRDL2 protein and the recombinant BMP2 protein. a: BMP-2 IP with anti-CHRDL2; b: CHRDL2 IP with anti-CHRDL2; c: mixture of BMP2 and CHRDL2; d: mixture of BMP2 and CHRDL2 IP with anti-CHRDL2. **D**. CHRDL2 inhibited the Smad1/5 phosphorylation of HCT116 cell induced by BMP2. The cytoplasm and nucleus protein extracts from HCT116 cells treated with BMP2, CHRDL2 or both for 1h were probed for p-Smad1/5, Smad1. **E**. Immunofluorescence array for p-Smad1/5 and Smad4 in HCT116 cell. Merged images of HCT116 cells treated with different concentrations of BMP2, CHRDL2 or both for 1 h, stained with DAPI and immunofluorescence stained with p-Smad1/5 and Smad4 respectively. Corresponding anti-rabbit secondary antibodies were conjugated with Alexa Fluor 488 and 555.

To identify cytokines that might interact with CHRDL2 in CRC cells, we performed co-IP assays to analyze the direct interactions between CHRDL2 and BMP2, BMP4, BMP6. As shown in Figure 6A and 6B, CHRDL2 could bind secreted mature forms of BMP2, BMP4, and BMP6 in cell culture media. To confirm this, we performed additional co-IP assays to determine whether recombinant CHRDL2 protein interacted with recombinant BMP2 protein. Our results revealed that CHRDL2 could bind the mature form of recombinant human BMP2 protein (Figure 6C). We then used western blotting to measure p-Smad1/5 levels, which are indicative of active BMP2 signaling. As shown in Figure 6D, adding100ng/ml BMP2 increased p-Smad1/5 levels in the cytosol and nucleus, and this effect was inhibited by CHRDL2 in a dose-dependent manner. We used immunofluorescence to determine the nuclear localization of p-Smad1/5 and Smad4. When BMP2 was added, p-Smad1/5 in the cytosol of HCT116 cells increased, and p-Smad1/5 as well as Smad4 began to translocate from the cytosol to the nucleus (Figure 6E). Again, CHRDL2 inhibited these processes. Therefore, CHRDL2 suppressed Smad1/5 phosphorylation and nuclear translocation by blocking the upstream stimulator BMP2.

### CHRDL2 could bind to Activin A, but could not affect the Smad2/3 signal

We performed co-IP assays to determine whether CHRDL2 binds to Activin A. In agreement with a previous study [22], we show in Supplementary Figure S2A and S2B, that CHRDL2 could bind to secreted mature forms of Activin A in cell culture media. In addition, we found that CHRDL2 could bind to mature recombinant human Activin A (Supplementary Figure S2C). We also used western blotting to check the levels of p-Smad2 and p-Smad3, indicators of active Activin A signaling. As shown in Supplementary Figure S2D, when 100ng/ml of Activin A were added, we detected no change in p-Smad2 and p-Smad3 levels in the cytosol or the nucleus. These data suggested that CHRDL2 could not affect p-Smad2 and p-Smad3 levels. We also used immunofluorescence to determine the localization of p-Smad2, p-Smad3 and Smad4. We found weak p-Smad2 expression and high p-Smad3 expression in HCT116 cells. Furthermore, Activin A and CHRDL2 did not affect the expression, the subcellular localizationor signaling of Smad2/3 (Supplementary Figure S2E).

### CHRDL2 blocked the effect of BMP2 on the proliferation inhibition and apoptosis inducing in CRC cells

**Figure 7:**
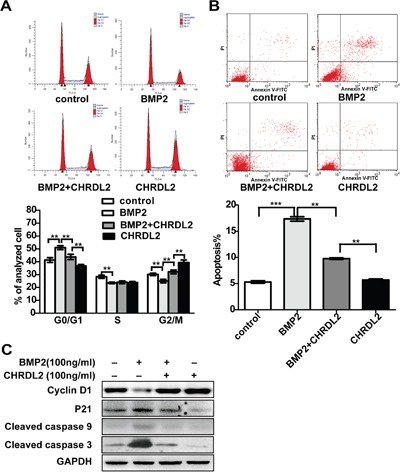
CHRDL2 blocks the BMP2 and attenuates the effect of promoting proliferation and inhibiting apoptosis induced by BMP2 in HCT116 cells **A**. Cell-cycle analysis by flow cytometry in HCT116 cells treated with 100 ng/ml BMP2 or 100 ng/ml CHRDL2. The histograms show the ratio of different cell phase populations (G0/G1, S and G2/M cells) in treated HCT116 cells. **B**. Apoptosis analysis by flow cytometry in HCT116 cells treated with 100 ng/ml BMP2 or 100 ng/ml CHRDL2. The histograms show the apoptosis rate in treated HCT116 cells. **C**. Western blot analysis was used to measure the P21, Cyclin D1, Cleaved caspase 9 and Cleaved caspase 3 in the lysis of HCT116 cells treated with 100 ng/ml BMP2 or 100 ng/ml CHRDL2.

To further investigate the influence of CHRDL2 on BMP2 and cell growth, we examined the effect of CHRDL2 on cell-cycle progression. Our cell cycle analysis showed that BMP2 induced cell-cycle arrest in the G0/G1 phase but the antagonist CHRDL2 promoted cell-cycle progression by increasing the number of cells in G2 phase (Figure 7A). Additionally, the cyclin-dependent kinase inhibitor p21 was upregulated and the cell cycle progression related protein Cyclin D1 was downregulated in HCT116 cell treated with BMP2 while CHRDL2 blocked such effects (Figure 7C). These findings suggested that CHRDL2 promoted CRC cell growth.

Previous studies showed that BMPs induced apoptosis in many tumor cells [23–26]. Therefore, we tested whether the antagonist CHRDL2 inhibited the apoptotic effect of BMP2 in CRC cells. HCT116 cells were treated with BMP2 for 48 h, with or without addition of CHRDL2. The cells were then harvested for apoptosis analysis by flow cytometry. Our results showed that BMP2 induced apoptosis, which was rescued by CHRDL2 (Figure 7B). In addition, we used western blotting to measure the levels of two apoptosis related proteins, caspase9 and caspase-3 (Figure 7C). We found that BMP2 significantly increased the rate of apoptosis (controls: 5.02 ± 0.66% versus BMP2: 17.27 ± 0.65%, *p*<0.01) while CHRDL2 counteracted this effect (BMP2: 17.27 ± 0.65% versus BMP2+ CHRDL2: 9.87±0.34%, *p*<0.01). These observations indicated that CHRDL2 protected CRC cells from apoptosis.

## DISCUSSION

BMP signaling has been shown to both suppress [26–31] and promote tumorigenesis [32–35]. TGF-β and BMPs have also been proposed to exert opposing tumor suppressor and oncogenic roles in cancer [36], depending on cancer type and cell type [37]. BMP pathway mutations have been proposed as one of the causes such functional changes [17, 38]. Previous studies have also demonstrated that BMPs could suppress [16, 17, 39] or promote [40, 41] tumorigenesis specifically in CRC. Interestingly, the tumor-promoting effects shown for BMP4 stemmed from mutations in BMP4 or Smads [38, 42, 43] while the tumor-promoting effects of BMP7 did not depended on Smad4 [44].

The dynamic balance between proliferation and differentiation of intestinal crypt stem cells depends on the regulation of Wnt, Notch, and BMP signaling [13, 14]. Loss of BMP signaling resulting for overexpression of BMP antagonists can also contribute to recurrence and metastasis [38, 45]. Such antagonists include Noggin [46, 47], Gremlin [48, 49], and Follistatin [50]. In this study, we analyzed the expression levels of the BMP antagonist CHRDL2 to assess whether it can be used as a prognostic marker in CRC. Our results here demonstrated that CHRDL2 was upregulated in CRC samples. Moreover, CHRDL2 levels correlated with clinical features, such as tumor size, TNM staging, and tumor differentiation, and all of these parameters were independent factors for CRC survival. This is consistent with previous observations that CHRDL2 expression was increased in breast, lung and colon cancers, suggesting that CHRDL2 promotes tumorigenesis [21]. Our survival rate and Cox proportional hazards model analyses showed that high CHRDL2 expression levels correlate with poor prognosis in CRC.

Although previous research has suggested that CHRDL2 may function as an oncogene in breast, lung and colon cancers, it is not yet clear how CHRDL2 promotes cancer cell proliferation and tumorigenesis in CRC. In this study, we showed that CHRDL2 promoted CRC cell proliferation *in vitro* and CRC tumorigenesis *in vivo*. Our immunohistochemistry results showed that CHRDL2 increased the expression of ki67 and cyclin D1, which were tumor proliferation markers.

Since the binding specificity of CHRDL2 to BMPs or Activin A is still controversial, with contradicting results being reported [20, 22], we studied the effects of CHRDL2 on BMPs or Activin A. We tested whether CHRDL2 blocked BMP ligands by incubating anti-CHRDL2 or BMP2, 4, 6 antibodies with HCT116 conditioned culture media followed by co-IP. Our results showed that CHRDL2 could bind BMP2, 4, 6. We further showed by co-IP that recombinant human CHRDL2 also interacted directly with recombinant human BMP2. These data suggested CHRDL2 can bind BMPs proteins. We then hypothesized that CHRDL2 might regulate the downstream Smad1/5 signaling pathways by binding and antagonizing BMPs. To test this hypothesis, we stimulated CRC HCT116 cell with BMP-2, with or without CHRDL2, and measured the phosphorylation levels of downstream signaling molecules smad1/5. Our results revealed that the effects of BMP2 increasing p-Smad1/5 were inhibited by CHRDL2. In addition, we performed immunofluorescence experiments and showed that the nuclear translocation of p-Smad1/5 and Smad4 was inhibited by CHRDL2. Taken together, these data suggest that CHRDL2 inhibits BMPs and their downstream signaling.

Activin A ligands can bind their receptor (ACVR) and activate Smad2/3, which then translocate into the nucleus. Here, we found that although CHRDL2 could bind to Activin A, neither Activin A or CHRDL2 affected the activation of Smad2/3 and the nuclear translocation of Smad4 in HCT116 cells. Previous studies have reported that ACVR is lost or mutated in primary colon cancer, and that the Activin A-ACVR-Smad pathway is inactivated in CRC [51–53].

BMPs are multifunctional proteins that regulate proliferation, differentiation, and other biological functions in cells [23, 54–57]. BMPs can also inhibit tumor proliferation and induce apoptosis [15, 16, 24, 26]. As an antagonist of BMPs, we hypothesized that CHRDL2 might inhibit apoptosis in CRC cells and promote proliferation. Consistent with previous observations that antagonists of BMPs promote cancer cell proliferation and inhibit apoptosis [9, 23, 24, 26, 54, 55, 57–59], our data showed that CHRDL2 inhibited apoptosis induction by BMP2 in HCT116 cells, furthermore, our study revealed that CHRDL2 enhanced proliferation of CRC cell by upregulating Cyclin D1 and downregulating P21.

Our findings suggest that CHRDL2 could be used as a novel biomarker for CRC. We also provide evidence that CHRDL2 acts as an oncogene in human CRC, highlighting it as a potential novel therapeutic target.

## MATERIALS AND METHODS

### Patients and samples

Primary CRC tumor samples were obtained from 125 colorectal cancer patients treated at Putuo Hospital Affiliated to Shanghai University of Traditional Chinese Medicine (Shanghai, China) between 2005 and 2009. Sixty corresponding adjacent normal tissues were obtained at the same time. The study was approved by the ethics committee of Putuo Hospital. Written informed consent was obtained from all patients.

### Cell lines and cultures

The HCT8, HCT15, HCT116, SW403, SW480, SW620, HT29, and LoVo cell lines were purchased from the Institute of Cell Biology (Shanghai, China). These cell lines were cultured in RPMI 1640 medium (Sigma). All media were supplemented with 10% fetal bovine serum (FBS, Gibco). All cell lines were maintained in a 5% CO2-humidified atmosphere at 37°C.

### Antibodies and reagents

Anti-CHRDL2 antibody (MAB2448) for western blot, anti-CHRDL2 antibody (AF2448) for immunohistochemistry, and HRP conjugate anti-mouse secondary antibodies (HAF007) were purchased from R&D. Anti-BMP2 (18933-1-AP), anti-BMP4 (12492-1-AP), anti-Activin A (60015-1-ig), and anti-BMP6 (60015-1-1g) antibodieswere obtained from Proteintech (China). Anti-Cyclin D1 (2978), anti-GAPDH (2118), anti-β-actin (12620), anti-Cleaved Caspase-9 (7237), anti-Cleaved Caspase-3 (9664), anti-Phospho-Smad1/5 (Ser463/465) (9516), anti-Smad1 (6944), anti-Smad4 (9515), anti-Smad2 (3122), anti-Phospho-Smad2 (Ser465/467) (11958), anti-Phospho-Smad3 (Ser423/425) (8828), anti-Smad3 (9513), anti-P21 (2947) and HRP Conjugate anti-rabbit secondary antibodies (7075) were obtained from Cell Signaling Technology Inc. (USA). Alexa Fluor 488 conjugated anti-rabbit secondary antibodies (P0176) and Alexa Fluor 555 conjugated anti-rabbit secondary antibodies (P0179) were obtained from Beyotime (China). Purified recombinant protein of Homo sapiens CHRDL2 (TP320245) and BMP2 (TP750012) were purchased from ORIGENE (USA).

### Cloning of CHRDL2 gene open reading frame

Five pairs of colorectal cancer tissues were used for CHRDL2 gene expression analysis. Total RNA was isolated from tissues using RNAiso Plus (Takara, Dalian, China), and First-strand cDNAs were prepared using the Primpscript RT reagent kit (Takara, Dalian, China). RT-PCR was performed using a Bio-Rad icycler and TaKaRa Taq HS Perfect Mix (Takara, Dalian, China). The CHRDL2 gene cDNA cloning primers used were: Forward, 5’-TCACCGAAGCTTATGGTTCCCGAGGTGAGG-3’; Reverse, 5’-TCACCGGGTACCGGTCTTTGTTATGTCTTGGTC-3’. PCR conditions were: 95°C for 5 min, followed by 30 two-step cycles (95°C for 40 s, 60°C for 40 s and 72°C for 60 s). RT-PCR products were analyzed on agarose gels and verified by DNA sequencing.

### Immunohistochemistry assay

All tissue specimens were formalin-fixed, embedded in paraffin, sectioned serially to 5 μm thickness slabs and mounted on glass slides. The reagents in subsequent process were purchased from Maixin Bio (FuZhou, China). Sections were incubated for 10 min in peroxidase blocking agent, washed for 3 min three times with phosphate-buffered saline (PBS), blocked with rabbit serum for 60 min at room temperature (RT), then incubated with antibody at 4°C overnight. The sections were washed three times with PBS, incubated with biotin conjugate rabbit anti-goat secondary antibody for 10 min at RT, washed again with PBS, incubated with HRP conjugated second antibodies for 10 min at RT, then developed with diaminobenzidine solution (DAB) and counterstained with hematoxylin. Meanwhile, serial sections were HE stained. Each section was read independently by two pathologists. CHRDL2 expression was semi-quantitatively scored based on the intensity of staining: (0) negative; (1) weak (light yellow); (2) moderate (yellow) and (3) strong (brown), and on the percentage of positive cells: (0) unstained; (1) ≤ 25%; (2) 26–50%; (3) 51–75%; and (4) > 75% positive cells.

### Flow cytometry

HCT116 cells were plated in six-well plates at a density of 2×10^5^ cells/well with cell culture medium and 10% fetal bovine serum (FBS). HCT116 cell were stimulated with BMP2 (100 ng/ml), CHRDL2 (100 ng/ml), BMP2 (100 ng/ml) + CHRDL2 (100 ng/ml) for 48 h. Subsequently, the cells were harvested by trypsinization, washed once with cold PBS. To investigate cell-cycle progression, these cells were fixed in ice-cold 70% ethanol and stored at 4°C for 1 h. After being washed once with cold PBS, the cells were subsequently stained for 30 min at RT in a solution containing 50 μg/ml PI (propidium iodide) and 10 μg/ml RNase A. Finally, flow cytometry analysis was performed using FACS Calibur system (Becton Dickinson, USA). PI was activated by a 488 nm laser, detected at 585 nm, and the data were recorded by Cell Quest software. The percentages of cells in various phases of the cell cycle were analyzed using ModFit software. To investigate apoptosis, cells were stained with PI and anti-annexin-V antibody (Becton Dickinson, USA) at 4°C for 1 h. Subsequently, cells were washed once with PBS and analyzed by FACS.

### Immunofluorescence array

HCT116 cells were seeded on cover slips pre-coated with 0.01% poly-lysine at a density of 5000 cells per well in a 24-well chamber. The cells were treated with or without BMP2, CHRDL2, fixed in 4% paraformaldehyde for 20 min at RT, permeabilized for 10 min in a 0.1% Triton X-100 followed by incubating in blocking buffer (Beyotime, China) (with 5% BSA) for 60 min. Cells were subsequently washed three times with PBS, incubated with primary antibodies at 4°C. Then the primary antibody was removed and the cells were washed five times with PBS. Alexa Fluor 488 and 555 conjugated anti-rabbit secondary antibodies were respectively added to the cells for 1 h. The slides were finally washed three times to remove unbound secondary antibody. Cell nuclei were stained by DAPI prior to being observed using a Zeiss LSM 700 confocal microscope.

### Quantitative real-time PCR assay

In all samples, sixty pairs of primary CRCs and their corresponding adjacent normal tissues were used for Quantitative real-time PCR (QRT-PCR) assay. Total RNA was isolated from tissues using RNAiso Plus, and the first-strand cDNAs was prepared using Primpscript RT reagent kit. QRT-PCR was performed using SYBR® Premix Ex Taq™ (Takara, Dalian, China) on CFX96 Real-time PCR System (Bio-Rad, USA). The following CHRDL2 gene primers were used: Forward, 5’-AAGATGAGGAAACTGAGGCT-3’, Reverse, 5’-GGTCACTTTGTCTGGACTGGC-3’. GAPDH served as a control, F: 5’CATCCTGCTCCTCATCATCTTTC-3’, R: 5’-TCCGTGTTCAGCTGCGGTA-3’. Each assay was performed in triplicate. PCR conditions were as follows: 95°C for 5 min, followed by 40 two-step cycles (95°C for 20 s and 60°C for 30 s). The fold change for CHRDL2 gene expression level was analyzed by the 2-ΔΔCT method [ΔΔCT = (CTCHRDL2 − CTGAPDH)_tumor_ − (CTCHRDL2 − CTGAPDH)_normal_]. All experiments were performed in triplicate.

### Lentivirus and transduction

The human *CHRDL2* gene shRNA lentivirus was purchased from GENECHEM (Shanghai, China). The lentivirus vector system is composed of the vectors GV248-GFP/Puro, pHelper1.0 (gag/pol element) and Helper2.0 (VSVG element). Three targeting sequences of the shRNA were designed and inserted into GV248-GFP/Puro vector. The *CHRDL2* shRNA target sequences were shRNA#1: AAGTCAGGAAGCAAGACTT; shRNA#2: AACATAAGAAAGCCTGTGT; shRNA#3: AAGTGAGCAATCGGATGAA. The human *CHRDL2* gene overexpressing lentivirus was purchased from GenePharma (Shanghai, China). The lentivirus vector system is composed of the vectors pGLV4-EF1a-EGFP and three packaging plasmids (pGag/Pol, pVSV-G, pRev). The cells were infected with lentivirus at an MOI of approximately 100 for 24 h. Cells were then switched back into normal culture media.

### Cell proliferation assay

The cultured cells were plated in a 96-well plate at a density of 3×10^3^ cells/well with 10% FBS medium. MTT assay was performed for cell proliferation after 24 h, 48 h, 72 h, and 96 h. Cell culture medium solution containing 5 mg/ml MTT was added to each well for 4 h. The supernatant was removed from cultured cells and 150 μl DMSO were added to each well. Absorbance was read at 570 nm using a plate reader (Bio-Rad). Each sample was run in six wells and experiments were repeated three times. Soft agar colony formation was performed for cell proliferation assay in six-well plates. The low melt agar was purchased from Bio-Rad. The base layer was made up of 0.6% agar, 1× medium and 10% FBS. The upper layer contained 200 cells, 0.35% agar, 1× medium and 10% FBS. Cells were cultured at 37°C for two weeks. Colonies were visualized by microscopy. Soft agar colony formation assays were performed in triplicates for each cell line. EdU (5-Ethynyl-2-deoxyuridine) staining was performed for cell proliferation assay in a 24-well chamber. The Cell-Light™ EdU DNA Cell Proliferation Kit (Ribobio, Guangzhou, China) was used to measure the proliferation rate of the cells according to the manufacturer's protocol. The percentage of EdU-positive cells represents the S-phase population and the cell's proliferation ability.

### Western blot and co-immunoprecipitation analysis

The cells were lysed with M-PER Mammalian Protein Extraction Reagent with Protease inhibitor cocktail (100×) (Sangon biotech, china) and 1 mM PMSF. The lysate were centrifuged at 4°C at 12000 g for 15 min and the supernatant was used for western blot. The protein concentration of measurement was done with the Bradford Coomassie Blue G-250 Method. Forty micrograms of protein were mixed with 5×SDS sample buffer and denatured by boiling for 10 min. Denatured protein loaded onto 10% polyacrylamide SDS gels (PAGE-SDS) and transferred onto PVDF membranes (Millipore). Membranes were blocked in 5% BSA for 2 h followed by incubation with primary antibodies overnight at 4°C. After washing three times for 10min in TBST, membranes were incubated with the HRP-conjugated secondary antibody for 2 h at RT. Subsequently, the membranes were washed again three times for 10 min in TBST and visualized using ECL Western Blotting Detection System (Millipore). The ratio of optical density of the bands was measured by a gel image analysis system (Bio-Rad) and normalized to β-actin. Co-immunoprecipitation (Co-IP) analysis was used to demonstrate direct interactions between CHRDL2 and BMP2, BMP4 and BMP6. The culture media for HCT116 cells was concentrated 10-fold by ultrafiltration through an ultra filtration device (NMWL, 3kDa) (Millipore Amicon® Ultra, UFC900396). Before co-IP, concentrated culture media were pre-cleared by incubating with normal IgG for 3h at 4°C. These samples were incubated with anti-CHRDL2, anti-BMP2, anti-BMP4, anti-BMP6 and normal IgG antibodies overnight at 4°C, then incubated with protein A+G agarose beads for 2h at 4°C. After being washed five times, protein A+G agarose beads were mixed with SDS sample buffer, denatured by boiling for 10min and loaded onto 10% PAGE-SDS for western blotting with corresponding antibodies. The co-IP analyses of recombinant human CHRDL2 protein and recombinant human BMP2 proteins were done similarly.

### Xenograft tumor growth assay

For the *in vivo* xenograft tumor growth assay, male nude mice [BALB/c nu/nu, 5-week-old, purchased from SLAC (Shanghai laboratory animal center, China)] were weighed and divided into five groups with ten animals/group: (1) HCT116/sh control (cont); (2) HCT116/shRNA#1; (3) HCT116/shRNA#3; (4) HCT8/cont; (5) HCT8/CHRDL2. Two million cells in 0.1 ml of PBS were injected into subcutaneous tissues of each mouse. The volume of xenograft tumors was measured every week, and the tumor volume was calculated using the following equation: V = ab^2^/2 (V = volume; a = length; b = width). Six weeks after injection, the tumor bearing mice were sacrificed and the tumors were excised and weighed.

### Statistical analysis

Data were analyzed with single factor analysis of variance and a Student's t-test using SPSS 17.0 software. These data were represented as mean ± SD. *P*< 0.05 was considered statistically significant. Survival rate analysis was performed using the Kaplan-Meier method. The univariate and multivariate Cox proportional hazards model analysis were performed to identify factors associated with disease-free survival (DFS) and overall survival (OS). Multivariate analyses were performed to reduce the confounders’ impact. These results were described by means of HR ratios (HRs) together with 95% confidence intervals (CIs), and *P* values were based on the Wald test.

## SUPPLEMENTARY MATERIALS FIGURES AND TABLES


