# Supplementary material for: Overexpression of colorectal cancer oncogene CHRDL2 predicts a poor prognosis
Source: Oncotarget. 2016 Dec 20;8(7):11489–506. doi: 10.18632/oncotarget.14039 (PMC5355280; doi:10.18632/oncotarget.14039)
Supplement: Supplementary file 1 [file oncotarget-08-11489-s001.pdf]

Overexpression of colorectal cancer oncogene CHRDL2 predicts a poor prognosis

SUPPLEMENTARY FIGURES

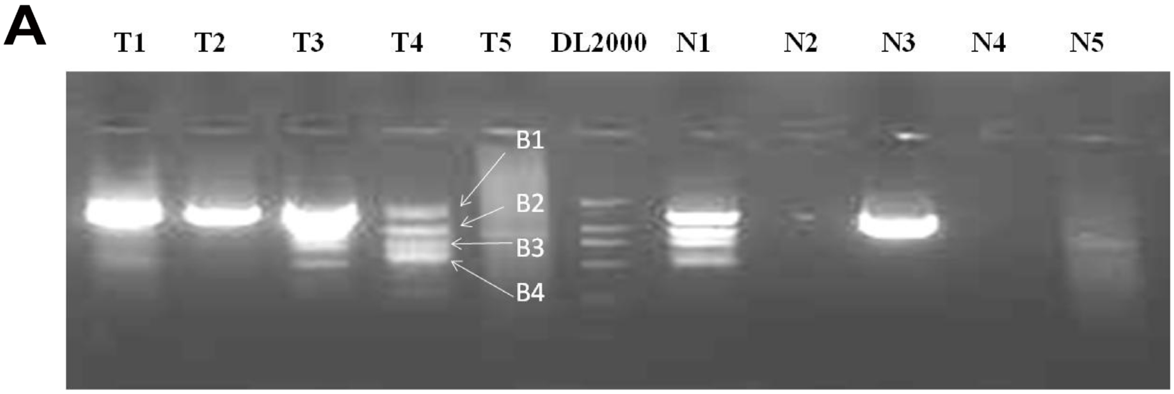

B

```
B1          MVPEVRVLSSLLGLALLWFPLDSHARARPDMFCLFHGKRYSPGESWHPYLEPQGLMYCLRCTCSEGAHVSCYRLHCPPVHCPQPVTEPQQCCPKCVEPHTPSGLRAPPKSCQHNGTMYQH  120

B3          MVPEVRVLSSLLGLALLWFPLDSHARARPDMFCLFHGKRYSPGESWHPYLEPQGLMYCLRCTCSEGAHVSCYRLHCPPVHCPQPVTEPQQCCPKCVEPHTPSGLRAPPKSCQHNGTMYQH  120

AY279090.1  MVPEVRVLSSLLGLALLWFPLDSHARARPDMFCLFHGKRYSPGESWHPYLEPQGLMYCLRCTCSEGAHVSCYRLHCPPVHCPQPVTEPQQCCPKCVEPHTPSGLRAPPKSCQHNGTMYQH  120


B1          GEIFSAHELFPSRLPNQCVLCSCTEGQIYCGLTTCPEPGCPAPLPLPDSCCQACKDEASEQSDEEDSVQSLHGVRHPQDPCSSDAGRKRGPGTPAPTGLSAPLSFIPRHFRPKGAGSTTV  240

B3          ------------------------------------------------------------------------------------------------------------------------

AY279090.1  GEIFSAHELFPSRLPNQCVLCSCTEGQIYCGLTTCPEPGCPAPLPLPDSCCQACKDEASEQSDEEDSVQSLHGVRHPQDPCSSDAGRKRGPGTPAPTGLSAPLSFIPRHFRPKGAGSTTV  240


B1          KIVLKEKHKKACVHGGKTYSHGEVWHPAFRAFGPLPCILCTCEDGRQDCQRVTCPTEYPCRHPEKVAGKCCKICPEDKADPGHSEISSTRCPKAPGRVLVHTSVSPSPDNLRRFALEHEA  360

B3          ------------------------------------------------------------------------------------------------------------------------

AY279090.1  KIVLKEKHKKACVHGGKTYSHGEVWHPAFRAFGPLPCILCTCEDGRQDCQRVTCPTEYPCRHPEKVAGKCCKICPEDKADPGHSEISSTRCPKAPGRVLVHTSVSPSPDNLRRFALEHEA  360


B1          SDLVEIYLWKLVKGIFHLTQIKKVRKQDFQKEAQHFRLLAGPHEGHWNVFLAQTLELKVTASPDKVTKT  429

B3          -------------------------------------------EGHWNVFLAQTLELKVTASPDKVTKT  136

AY279090.1  SDLVEIYLWKLVKGIFHLTQIKKVRKQDFQKEAQHFRLLAGPHEGHWNVFLAQTLELKVTASPDKVTKT  429
```

Supplementary Figure 1: Identification of CHRDL2 gene sequence in colorectal cancer tissue. A. The DNA electrophoresis of *CHRDL2* gene PCR products from five pairs of colorectal cancer tissue and their matched normal tissues (N, normal tissue, T, tumor tissue). Four model bands were marked B1-B4. **B.** Comparison of B1, B3 sequence and CHRDL2 variant I (AY279090.1) protein sequence. The position of the signal peptide (SP) and three cysteine-rich repeats (CR) domains (CR1, CR2, CR3) are indicated.

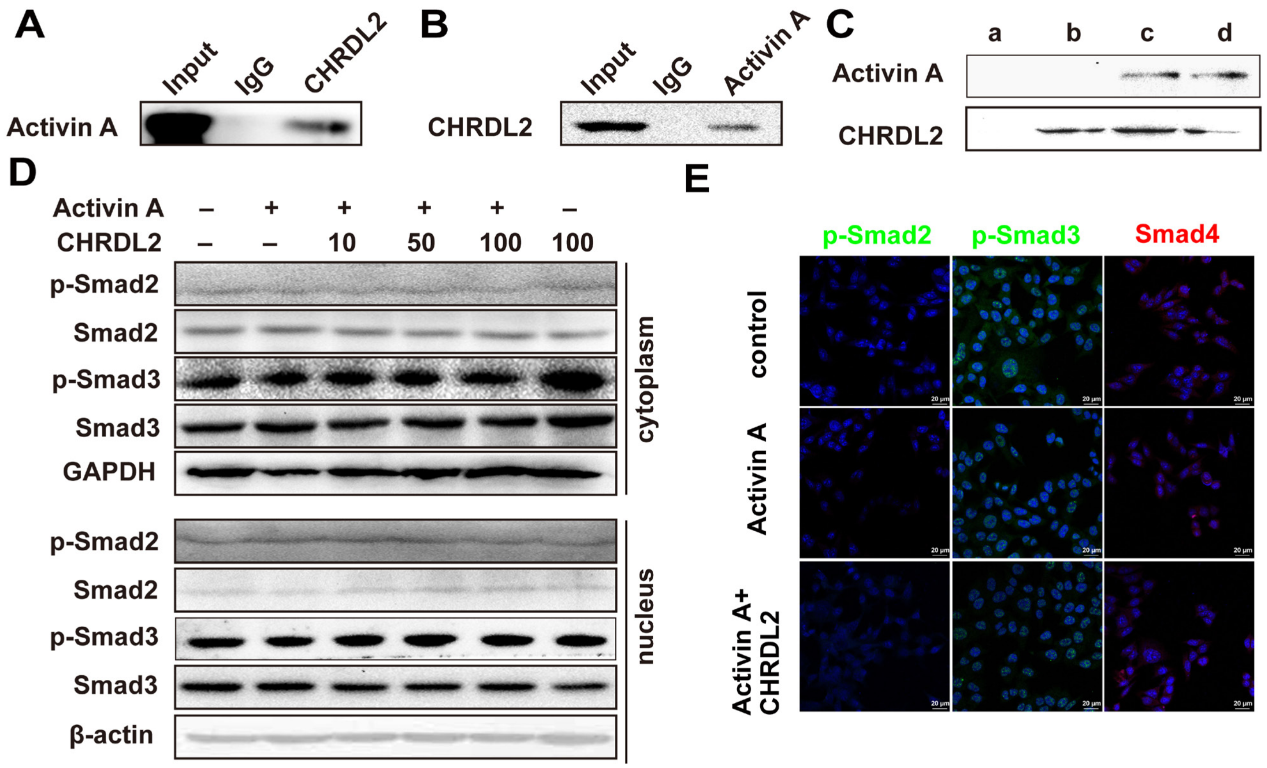

Supplementary Figure 2: CHRDL2 binds to Activin A but has not effect on the phosphorylation of Smad2 and Smad3. **A.** Co-IP of CHRDL2 and Activin A. HCT116 cell culture media were performed using anti-CHRDL2 and control (mouse IgG) antibodies. Immunoprecipitates and culture media were subjected to western blot analysis using anti-Activin A antibody. **B.** Same as in panel A, culture media were performed using anti-Activin A and control (rabbit IgG) antibodies, and western blot analysis using anti-CHRDL2 antibody. **C.** Co-IP of recombinant CHRDL2 protein and the recombinant Activin A protein. a: Activin A IP with anti-CHRDL2); b: CHRDL2 IP with anti-CHRDL2; c: mixture of Activin A and CHRDL2; d: mixture of Activin A and CHRDL2 IP with anti-CHRDL2. **D.** CHRDL2 can not affect the Smad2 and Smad3 phosphorylation of HCT116 cell induced by BMP2. The cytoplasm and nucleus protein extracts from HCT116 cells treated with Activin A, CHRDL2 or both for 1 h were probed for p-Smad2 and p-Smad3. **E.** Immunofluorescence array for p-Smad2, p-Smad3 and Smad4 in HCT116 cells. Merged images of HCT116 cells treated with different concentrations of Activin A, CHRDL2 or both for 1 h, stained with DAPI and immunofluorescence stained with p-Smad2, p-Smad3 and Smad4. Corresponding anti-rabbit secondary antibodies were conjugated with Alexa Fluor 488 and 555.
